# Acute Peritonitis Is Not Always a Surgical Fix: A Rare Case of Mixed Connective Tissue Disease Presenting as Polyserositis

**DOI:** 10.7759/cureus.36652

**Published:** 2023-03-24

**Authors:** Ariana R Tagliaferri, Abraam Rezkalla, Charissa Nichols, Prathyusha Chennupati, George Angelo Bellardini

**Affiliations:** 1 Internal Medicine, St. Joseph's Regional Medical Center, Paterson, USA; 2 Internal Medicine, St. Joseph's Univesity Medical Center, Paterson, USA; 3 Psychiatry, Bergen County Hospital, Bergen, USA

**Keywords:** peritonitis, acute abdomen, rheumatological disease, autoimmune disease, mixed-connective-tissue-disease, serositis, polyserositis

## Abstract

Mixed connective tissue disease (MCTD) is a complex rheumatologic condition whose diagnosis often presents a challenge to even specialists in the field. Many cases are therefore underrecognized or misdiagnosed due to the heterogeneity of the presentation and manifestations. This report highlights the intricacies of diagnosing a case of MCTD when the presenting symptom is atypical. Herein, we present a case of a young girl who had severe abdominal pain, initially concerning for acute peritonitis from cholecystitis, and was found to have polyserositis affecting the pleural space, pericardium, peritoneum and pelvis secondary to mixed connective tissue disease and adrenal insufficiency.

## Introduction

Mixed connective tissue disease (MCTD) is a rheumatological autoimmune disease, also known as Sharp’s syndrome [1,2]. The disease is characterized by an array of non-specific systemic symptoms that overlap with those of other autoimmune diseases, such as systemic lupus erythematosus (SLE), scleroderma, rheumatoid arthritis (RA), and/or polymyositis, and thus, the main diagnostic feature is the presence of autoantibodies called anti-U1 ribonucleoprotein (RNP) [1]. Human leukocyte antigen (HLA) molecules and T-cell receptors generate anti-U1-RNP complexes, and thus genetics and inflammatory cascades play a major role in the pathogenesis; however, ultimately, the cause is unknown [3]. MCTD occurs mostly in females between the ages of 30 and 39 years old, with women between the ages of 50 and 65 following close behind [1,2]. There are limited data regarding the epidemiology of MCTD and it is likely underreported, but a nationwide study from Norway in 2011 reported a prevalence of 3.8 per 100,000 adults and an incidence of 2.1 million patients diagnosed annually [1].

Although MCTD affects all races and the clinical manifestations are not different based on ethnicity, the manifestations of MCTD are very broad and non-specific [3]. It is debated as to whether MCTD is a separate phenomenon or if the disease is an early phase of another autoimmune disease that becomes more apparent over time [2]. This is because particular features are likely to appear first, such as Raynaud’s syndrome, arthritis, and puffy hands, whereas other symptoms, such as sclerodactyly, motility disorders, and interstitial lung disease appear much later [1]. In fact, Raynaud’s syndrome may be the only clinical sign for many years prior to diagnosis [1]. Rheumatoid factor and other autoantibodies may be positive in some patients at either the time of initial presentation and/or time of final diagnosis [4].

Because of the clinical heterogeneity, the diagnosis may be challenging, but prior studies have shown that the predominating symptom in each individual will remain active as the predominant symptom throughout their lifetime [5]. The disease is also milder than SLE or scleroderma [5]. There are no clear guidelines outlining the treatment of MCTD, but the majority of clinical manifestations and organs involved can be adequately treated with steroids [3]. Hydroxychloroquine, intravenous immunoglobulins, or methotrexate are also used in the treatment of MCTD, particularly for hematological or dermatological manifestations [1]. Although the prognosis is contingent on the organ systems involved, prior studies have not shown a mortality difference compared to the general population [3,4].

Herein, we present a case of a young girl who had severe abdominal pain, initially concerning for acute peritonitis from cholecystitis, and was found to have polyserositis affecting the pleural space, pericardium, peritoneum, and pelvis secondary to mixed connective tissue disease and adrenal insufficiency.

## Case presentation

A 24-year-old female with no significant past medical history presented to the emergency department with complaints of severe and diffuse abdominal pain, most prominent throughout the lower quadrants. The pain was sharp and non-radiating and came on suddenly at rest. The patient also complained of fever, chills, and nausea for the three days prior to admission. Through further history, her last menstrual cycle was 20 days before the presentation. She was sexually active with one male partner and reported the use of protection intermittently. She had never been tested previously for sexually transmitted infections (STI). She had no personal or family history of gallstones, was not pregnant, and she was not overweight.

**Figure 1:**
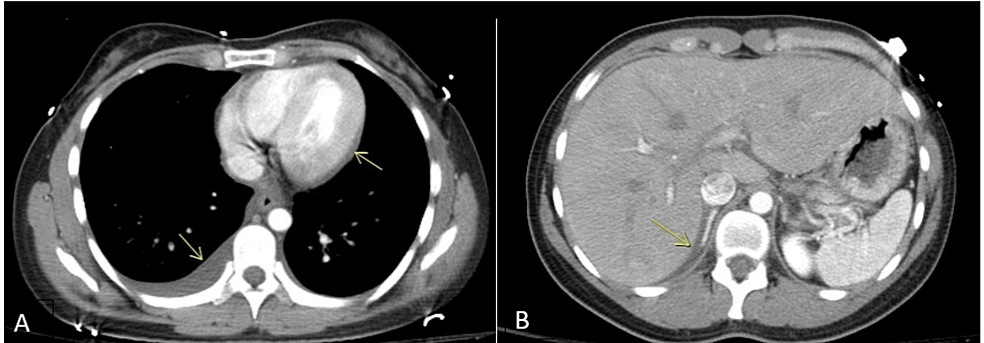
A computerized tomography of the abdomen and pelvis demonstrating pleural effusions and intra-abdominal fluid. (A) Sagittal views of a CT demonstrating a small right basilar pleural effusion, as denoted by the yellow arrow, as well as a pericardial effusion, which is also denoted by another yellow arrow. (B) Sagittal views of a CT demonstrating a small amount of perihepatic fluid, as denoted by the yellow arrow.

On arrival, she was afebrile with a blood pressure of 85/54 mmHg and a heart rate of 100 beats per minute. On the physical exam, the patient was in severe acute distress, and she was lying very still in the hospital bed, afraid to move due to pain. The patient had no scleral icterus or conjunctival pallor. She had a firm abdomen with severe tenderness on minimal palpation, associated with rebounding and guarding. Her bowel sounds were normoactive, and no organomegaly was appreciated. Murphy's sign was positive, although this finding is not specific to this presentation of acute abdomen. Laboratory studies revealed a normal complete metabolic panel as well as a complete blood count. Both troponin levels (68 pg/mL; reference range 3-17 pg/mL) and lactate dehydrogenase (LDH; 291 units/L; reference range 140-271 units/L) were noted to be elevated. Thyroid-stimulating hormone (TSH) and free T4 were also within normal limits. Urinalysis and an STI screen were negative, including human immunodeficiency virus (HIV). A surgical evaluation was prompted at this time. A computerized tomography (CT) of the abdomen and pelvis with intravenous contrast was performed, revealing a small right-sided basilar pleural effusion, as well as pericardial effusion, a small amount of perihepatic fluid, and a thickened gallbladder wall, was thickened (Figure 1).

**Figure 2:**
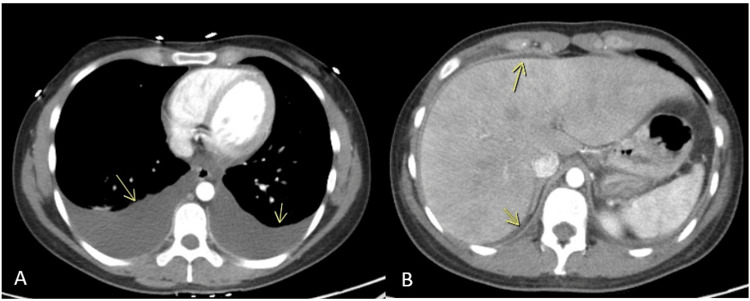
A computerized tomography of the abdomen and pelvis demonstrating interval increase in ascites and pleural effusions. (A) Sagittal views of a CT demonstrating moderate bilateral pleural effusions, right more than left, significantly increased from the prior examination. The pleural effusions are indicated by the arrows. (B) Moderate volume ascites noted in the abdomen and pelvis increased in volume since the prior exam, as indicated by the yellow arrows.

There were also findings of a complex hemorrhagic and cystic lesion of the right adnexa with free pelvic fluid, concerning an ovarian cyst rupture. The gynecology team performed a transvaginal pelvic ultrasound, which redemonstrated the right ovarian hemorrhagic cyst measuring approximately 3.4 cm. A right upper quadrant abdominal ultrasound (US) suggested gallbladder wall thickening with pericholecystic fluid without the presence of gallstones. There was sonographic evidence of Murphy’s sign. At this stage, given the concerns about her hemodynamic instability and peritoneal signs, the working diagnosis was peritoneal inflammation secondary to ruptured ovarian cysts versus acalculous cholecystitis. However, the diagnosis was unclear. This prompted an autoimmune workup, which resulted a few days later. Given the hypotension and pericardial effusion, cardiology also performed an urgent bedside transthoracic echocardiogram (TTE), which confirmed the presence of a small pericardial effusion without right ventricular diastolic collapse. Cardiac tamponade was excluded at this time. The patient’s hemoglobin remained stable at 13 mg/dL throughout the first 24 hours of her admission, despite being persistently hypotensive. Thus, the focus was shifted to her cholecystitis, for which surgical intervention was initially deferred due to a negative hepatobiliary iminodiacetic acid (HIDA) scan, and the absence of leukocytosis or abnormal liver enzymes. The surgical team felt that cholecystitis was not the cause of her pain or hypotension, and they could not explain the polyserositis with this finding alone. However, she remained hypotensive with a declining mean arterial pressure despite intravenous crystalloid boluses and additionally complained of increased abdominal girth and pain. For this, a CT of the abdomen and pelvis was repeated with intravenous contrast, demonstrating an interval increase in the size of the pleural effusions, pericardial effusions, and intra-abdominal and pelvic fluid collections (Figure 2).

Given this information, she was taken to the operating room, where the surgical team found complex bilateral ovarian cysts and a moderate amount of clear ascitic fluid in the peritoneal cavity, which was sent for pathology. The peritoneal fluid pathologically showed mostly bland-appearing mesothelial cells with a few scattered acute and chronic inflammatory cells. There were no malignant cells identified pathologically. The remaining fluid was suctioned intra-operatively. The cysts were not hemorrhagic at that time. No further intervention was warranted. The gallbladder was edematous but there was no evidence of cholecystitis and it was subsequently not removed. During the first 72 hours of admission, the patient was noted to have multiple random blood glucose readings in the 50s and 60s (mg/dL), prompting an adrenal and autoimmune workup (Table 1).

**Table 1:** An autoimmune panel and adrenal insufficiency investigations. A table outlining the autoimmune panel and adrenal insufficiency investigations, which were sent after repeated episodes of hypoglycemia and persistent hypotension. Reference ranges are included.

Laboratory study	Reference range	Laboratory value
Antinuclear antibody	<0.180 (neg)	>0.180
Speckled pattern	N.A	1:320
Anti-double-stranded deoxyribonucleic acid (DNA)	0–9	1
Anti-Jo1 antibody	0.0–0.9	<0.2
Anti-chromatin antibody	0.0–0.9	<0.2
Anti-ribosomal antibody	0.0–0.9	<0.2
Centromere B antibody	0.0–0.9	<0.2
Anti-U1 ribonucleoprotein antibody (RNP)	0.0–0.9	5.9
Scleroderma-70 Antibody	0.0–0.9	<0.2
Sjogren syndrome (SS) A and SS B antibodies	0.0–0.9	<0.2
Smith antibody	0.0–0.9	<0.2
Lupus anticoagulant antibody	N.A	Negative
Adrenocorticotrophic hormone (ACTH)	7.2–63.3 pg/mL	72 pg/mL
Cortisol (morning)	4.3–22.4 mcg/dL	1.1 mcg/dL

Both adrenocorticotropic hormone (ACTH) (72 pg/mL) and morning cortisol (1.1 mcg/dL) levels confirmed primary adrenal insufficiency. Additionally, the autoimmune panel was significant for a positive antinuclear (ANA) immunofluorescence with a speckled pattern titer of 1:320 detecting the RNP antigen. She was diagnosed with mixed connective tissue disease solely on the basis of RNP positivity, as she did not meet the Alarcon-Segovia criteria; however, many patients do not meet the criteria at the time their first symptoms appear. She was seen by endocrinology and treated with intravenous hydrocortisone for three days for a likely adrenal crisis. On day 5 of hospitalization, her pain and abdominal swelling improved, and she was discharged to follow up with rheumatology.

## Discussion

The Alarcon-Segovia criteria are often used to aid in the diagnosis of MCTD, but there is currently no tool to measure disease activity [1,5]. As MCTD is a heterogeneous disease with nonspecific symptoms, it is estimated that only 10-33% of patients will fulfill the Alarcon-Segovia criteria at the time their first symptom appears [1,2]. In fact, many patients will only have one predominant symptom for up to 10 years prior to diagnosis, and 90% of patients are diagnosed with other conditions first [1,2,4]. Prior studies have shown that Raynaud’s phenomenon is the most common presenting symptom, but symmetric arthralgias are the most predominant symptom by the time patients fulfill the Alarcon-Segovia criteria [1,2,4]. Puffy fingers are seen in approximately 60% of patients at the time of fulfillment, and following this are symptoms of poor gastric motility, myositis, and heartburn [1,4]. Our patient did not meet the criteria and was diagnosed exclusively by her seropositivity.

Less commonly, there are symptoms of lung fibrosis, hypocomplementemia, and hematological abnormalities [2,4]. Sclerodactyly is a late finding, and the prevalence of this doubles from the time of the initial symptom to the fulfillment of criteria [4]. Serositis, or inflammation of serous membranes, is one of the least common symptoms and manifests late in the disease [4,6]. Although it can affect any organ system, it usually develops in one organ system at a time [4]. This was the case in 50 patients diagnosed with MCTD who were followed for 10 years after diagnosis, in which six total patients developed serositis late in follow-up, affecting the pericardium or pleural space [4]. Our patient presented at a young age with polyserositis or inflammation with effusion of different serous membranes, affecting the pericardium, pleural space, peritoneal space, and intra-abdominal space [6]. This is a novel presentation since polyserositis normally manifests late in the disease.

Polyserositis itself has a wide range of etiologies, from infectious to autoimmune diseases, but the most common cause is a neoplasm [6]. In many cases, the cause remains unknown [6]. Antinuclear antibodies and adenosine deaminase usually differentiate autoimmune disease from neoplasms, in which only LDH is high [6]. Polyserositis has been described in SLE, adult-onset still disease, and familial Mediterranean fever, but nonetheless, the most common locations are in the pericardium and pleural cavities in up to 83% of patients [6]. Rarely is polyserositis described in the setting of MCTD and moreover manifests in the abdomen and pelvis, such as in our patient. To the best of our knowledge, only one other case reports a patient with MCTD who developed abdominal polyserositis; however, this case was induced by Salmonella gastroenteritis [7]. It was thought that the infection precipitated a pathway of circulating immune complexes, which were deposited into the affected tissues, such as the gastrointestinal tract [7]. The two findings of adrenal insufficiency and MCTD make our case particularly unique. The hypotension and hypoglycemia were likely the patient’s baseline, which further confounded our diagnosis. There are other cases in the current literature describing polyserositis in the setting of infection, although this was not the case for our patient, and cases describing polyserositis with adrenal insufficiency in the setting of polyglandular autoimmune syndromes (PGA) [8].

PGA are a constellation of autoimmune diseases that affect more than two endocrine glands as well as non-endocrine organs [8]. Patients may have immune disorders such as Sjogren’s, scleroderma, SLE, thyroid disease, pernicious anemia, thrombocytopenia, celiac disease, diabetes, or parathyroid disease, just to name a few [8]. Primary adrenal insufficiency predominates in type II PGA [8]. Both type I and type II may present with polyserositis, most commonly affecting the cardiovascular and respiratory systems [8]. Although our patient did not demonstrate clinical stigmata or laboratory evidence of diabetes or thyroid disease, an autoimmune syndrome such as PGA type II cannot be excluded in a patient with adrenal insufficiency and mixed connective tissue disease.

## Conclusions

Many diagnostic challenges exist in MCTD and thus it is likely underreported. Clinical suspicion should remain high in a patient who presents with a constellation of non-specific symptoms or features of other autoimmune diseases; however, it is important to note that isolated symptoms may be evident in early disease stages. Our case was particularly difficult to diagnose because she did not have other systemic signs of MCTD, and her peritonitis with hypotension and hypoglycemia distracted us from her underlying adrenal insufficiency. Our case is particularly unique because MCTD rarely presents as polyserositis, and when serositis occurs, it is usually either in the pericardium or pleural space independently. Although MCTD co-exists with other rheumatological diseases, it is not documented in current literature to co-exist in someone with adrenal insufficiency.
